# CSTP1, a Novel Protein Phosphatase, Blocks Cell Cycle, Promotes Cell Apoptosis, and Suppresses Tumor Growth of Bladder Cancer by Directly Dephosphorylating Akt at Ser473 Site

**DOI:** 10.1371/journal.pone.0065679

**Published:** 2013-06-17

**Authors:** De-Xiang Zhuo, Xiao-Wei Zhang, Bo Jin, Zheng Zhang, Bu-Shan Xie, Cheng-Lin Wu, Kan Gong, Ze-Bin Mao

**Affiliations:** 1 The Department of Biochemistry and Molecular Biology, Health Science Center, Peking University, Beijing, China; 2 The Department of Urology, Peking University First Hospital and the Institute of Urology, Peking University, Beijing, China; 3 The Department of Laboratory Medicine, the First Hospital of Sanming Affiliated to Fujian Medical University, Sanming, Fujian, China; University of Pecs Medical School, Hungary

## Abstract

Akt/protein kinase B is a pivotal component downstream of phosphatidylinositol 3-kinase (PI3K) pathway, whose activity regulates the balance between cell survival and apoptosis. Phosphorylation of Akt occurs at two key sites either at Thr308 site in the activation loop or at Ser473 site in the hydrophobic motif. The phosphorylated form of Akt (pAkt) is activated to promote cell survival. The mechanisms of pAkt dephosphorylation and how the signal transduction of Akt pathway is terminated are still largely unknown. In this study, we identified a novel protein phosphatase CSTP1(complete s transactivated protein 1), which interacts and dephosphorylates Akt specifically at Ser473 site *in vivo* and *in vitro*, blocks cell cycle progression and promotes cell apoptosis. The effects of CSTP1 on cell survival and cell cycle were abrogated by depletion of phosphatase domain of CSTP1 or by expression of a constitutively active form of Akt (S473D), suggesting Ser473 site of Akt as a primary cellular target of CSTP1. Expression profile analysis showed that CSTP1 expression is selectively down-regulated in non-invasive bladder cancer tissues and over-expression of CSTP1 suppressed the size of tumors in nude mice. Kaplan-Meier curves revealed that decreased expression of CSTP1 implicated significantly reduced recurrence-free survival in patients suffered from non-invasive bladder cancers.

## Introduction

Bladder cancer is the second most common malignancy in the genitourinary tract, with 350,000 newly diagnosed cases and over 145,000 deaths each year worldwide [1]. Because long-term surveillances of the patients are necessary, together with possible tumor recurrences and other complications, treatments of bladder cancers usually cost a lot of money. According to the differences in clinical developments, pathology and molecular alteration profiles, bladder cancers are classified into two types of carcinomas: the non-muscle invasive superficial, papillary carcinomas and the muscle invasive carcinomas [2]. The non-muscle invasive superficial, papillary carcinomas are usually low life-threatening, but with high incidences and high recurrences, while the muscle invasive carcinomas often cause distant metastasis and quick deaths [3].

Multiple signaling pathways are implicated in initiation and progression of bladder cancers, including mutations in PI3K/Akt and Ras/MAPK oncogenic path way components and alterations in the tumor suppressors, such as p53 and Rb. There is increasing evidence indicating that alterations in these pathway components are not only associated with initiation of bladder cancers, but also strongly correlated with disease recurrence, progression and survival. For example, gain of function mutations of Ras, FGFR3, PIK3CA are frequently implicated in the non-muscle invasive superficial, papillary carcinomas, whereas loss of function alterations of p53 and Rb genes are found more frequently in aggressive, muscle invasive carcinomas[4]–[6].

The phosphatidylinositol 3-kinase(PI3K) pathway regulates the balance between cell survival and apoptosis, and this balance is often disrupted in many types of human cancers, including the human urothelial bladder cancers [2]. Akt is a pivotal downstream component of the PI3K pathway, which can be activated by sequential phosphorylation at two sites conserved in the AGC kinase family [7]. For example, an upstream kinase PDK-1 can phosphorylate the Thr308 site in the activation loop of Akt1 [8], [9], which triggers the autophosphorylation of Akt1 at its C-terminal Ser473 [10], and thus fully activates Akt1. Akt signaling can be terminated by two mechanisms: removal of the active lipid second messenger that is catalyzed by the lipid phosphatase PTEN (phosphatase and tensin homolog deleted on chromosome ten) [11], and dephosphorylation of the activated Akt. Akt can also be directly dephosphorylated by the PP2A-type phosphatases [12] or other protein phosphatases such as PHLPP1(PH domain leucine-rich repeat protein phosphatase 1) and PHLPP2 [13], [14].

Here, we reported a novel protein phosphatase, termed the complete s transactivated protein 1(CSTP1) [15], whose expression was selectively reduced in non-muscle invasive bladder cancers. To make a further understand of the significance of CSTP1 in bladder carcinogenesis, in this study, we will go deep insight into i. the effects of CSTP1 on bladder cancer cell cycle, apoptosis and tumor formation in vivo and the underlined molecular mechanisms; ii. the molecular mechanisms through which CSTP1 exerts its biological function; iii. the significance of decreased expression of CSTP1 on the recurrence and prognosis of non-invasive bladder cancers.

## Materials and Methods

### Ethics Statement

This study was approved by the ethics committees of the Peking University First Hospital (Permit Number:2008 [96]), and written informed consent was obtained from each subject. All animal experiments were conducted in strict accordance with the recommendations in the Guide for the Care and Use of Laboratory Animals of the Health Science Center of Peking University. The protocol was approved by the Ethics Committee of Animal Experiments of the Peking University First Hospital (Permit Number:J201112).

### Patients

Eighty-six bladder cancer patients that were newly diagnosed in the Institute of Urology, Peking University were enrolled in this study. Among them, 62 cases had non-invasive bladder cancers and underwent organ-preserving after transurethral resection of bladder tumors (TURBT). The medium age (± SD) of the 62 patients was 65.3 ± 11.5 years old (40 men and 22 women).

### Cell Culture

RT4, SV-HUC1, EJ, and T24 cells were cultured in RPMI 1640 medium supplemented with 10% FBS, 100 U/ml penicillin, and 100 µg/ml streptomycin. Hela and 293T cells were cultured in DMEM medium supplemented with 10% FBS and 100 U/ml penicillin. Sf9 cells were maintained in Sf-900 II SF medium containing penicillin/streptomycin at final concentration (50 units/ml penicillin, 50 µg/ml streptomycin) at 27 oC.

### Real Time PCR

Total RNA was extracted by using TRIzol reagent (Invitrogen, Carlsbad, CA, USA) according to the manufacturer’s protocol. The first strand cDNA was synthesized using SuperScript TMIII first strand kits (Invitrogen, Carlsbad, CA, USA). GAPDH was used as an internal control. CSTP1_RT and GAPDH_RT primers used in this study are given in Table 1. The difference in expression of CSTP1 mRNA was calculated using the 2-ΔCt method described by Schmittgen [16].

**Table 1:** Oligonucleotides used in this study.

Oligonucleotides	Sequences
CSTP1_RT_F	5' ACATCCACTCAAACGCTCACC 3'
CSTP1_RT_R	5' ACACATGGGGATTATGGGGAT 3'
GAPDH_RT_F	5' ACGGATTTGGTCGTATTGGG 3'
GAPDH_RT_R	5' TGATTTTGGAGGGATCTCGC 3'
CSTP1_MYC_F	5' AA G GATC C AAC TCG CTC GCC ATG TCG 3'
CSTP1_MYC_R	5'AGAATTCCTTTTTTCTTGATCAAATCCATGAGATC 3'
CSTP1_GFP_F	5' AAGAATTCAACTCGCTCGCCATGTCG 3'
CSTP1_GFP_R	5'AAGGATCCTTTTTTCTTGATCAAATCCATGAGATC 3'
CSTP1_ZsG_F	5' AA ACTAGTGAAACTCGCTCGCCATGTC 3'
CSTP1_ZsG_R	5' AAGGATCCACGGGAAGGAGCGTCATTTT 3'
CSTP1ΔPP2Ac_F	5' AGGCCACTACCACAGGAATG 3'
CSTP1ΔPP2Ac_R	5' GGTTTGGGGTTCAGCTTGTT 3'
Akt_Tag_F	5' AAGGATCCATGAGCGACGTGGCTATTGTG 3'
Akt_Tag_R	5' AACTCGAGCCTCCAAGCTATCGTCCAGC 3'
Akt_ZsG_F	5' AACACGTCGCGGGAGCCTCGGGCAC 3'
Akt_ZsG_R	5' AAGGATCCTCCATCCCTCCAAGCTATCGTCCAG 3'
Akt_Mut_F	5' CTTCCCCCAGTTCGACTACTCGGCCAGCGGC 3'
Akt_Mut_R	5' GCCGCTGGCCGAGTAGTCGAACTGGGGGAAG 3'
CSTP1_pET_F	5' AAGGATCCATGTCGGCTGCAGAGGCG 3'
CSTP1_pET_R	5' AAAAGCTTGGAGCGTCATTTTTTCTTGATCAAA 3'
CSTP1_siRNA_target	5' GTCTAGATGAGCTGAGTGA 3'
CSTP1_siRNA_neg	5' TTCTCCGAACGTGGCACGA 3'
GAPDH_NB_F	5' AAAATCAAGTGGGGCGATG 3'
GAPDH_NB_R	5' GCCTGCTTCACCACCTTC T 3'
Akt_His_F	5' AAGAATTCTCGCGGGAGCCTCGGGCAC 3'
Akt_His_R	5' AAACCGGTGGCCGTGCCGCTGGCCG 3'
Akt_Bac_F	5' AAGGATCCGGCACCATGAGCGACGT 3'
Akt_Bac_R	5' AACTCGAGGGTTTAAACTCAATGGTGATGGTG 3'

### Animal Experiment

Six to 8 week-old female nude mice with BALB/c background were purchased from Peking University. The 5×106 of EJ cells over-expressing CSTP1 or CSTP1 ΔPP2Ac and control cells were injected subcutaneously into mice. The size of tumors was measured once a week with a caliper, and tumor volumes were calculated using the formula π/6 × length × width2. Each point represents the mean ± S.D. for different animal measurements (n = 6).

### Antibody Preparation

Anti-CSTP1 polyclonal antibody was prepared by immunizing rabbit with the 20-mer peptide, IDEDDDYYFNLSKSTRKKLA, and antiserum was purified by affinity chromatography.

### Plasmid Construction

The coding region of CSTP1 was obtained from normal bladder endothelial cDNA using primers CSTP1_MYC_F and CSTP1_MYC_R, and cloned into the pcDNA3.1 myc/his C vector to generate recombinant plasmid pcDNA3.1 myc/his C -CSTP1. All primers used in this study are given in Table 1. To generate GFP fusion construct, the entire coding region of CSTP1 was amplified using primers CSTP1_GFP_F and CSTP1_GFP_R, and subcloned into the pEGFP-N1 expression vector as pEGFP-CSTP1. The lentivirus expression plasmid pZsG-CSTP1 was constructed through inserting PCR product amplified by CSTP1_ZsG_F and CSTP1_ZsG_R into pZsG plasmid. pZsG-CSTP1 ΔPP2Ac was obtained using primers CSTP1ΔPP2Ac_F and CSTP1ΔPP2Ac_R and the products were ligated again using T4 DNA ligase. The coding region of Akt1 was amplified with primers Akt_Tag_F and Akt_Tag_R and cloned into pCMV Tag-2B plasmid. For construction of the Akt (S473D) phosphomimetic mutant, wild type Akt1 was first cloned into pZsG plasmid by using of PCR (primers Akt_ZsG_F and Akt_ZsG_R are given in Table 1). Point mutation in Akt1 (Ser473) that converts Ser to Asp was generated by using the QuikChange site-directed mutagenesis kit(Stratagene, La Jolla, CA ,USA) following the manufacturer’s instructions with the following primers (Akt_Mut_F and Akt_Mut_R, Table 1). For bacterial expression of CSTP1 protein, CSTP1 cDNA was cloned into pET-42a vector with primers (CSTP1_pET_F and CSTP1_ pET_R, Table 1).

### RNA Interference

The human CSTP1 siRNA target sequences (CSTP1_siRNA_target, Table 1) are relative to the first nucleotide of the start codon of the human CSTP1 coding sequence. A non-related, scrambled siRNA was used as the negative control (CSTP1_siRNA_neg, Table 1). The siRNA was synthesized by Shanghai Genechem Co., Ltd, China. CSTP1 knockdown was performed by transfection of siRNA into MCF cells using Lipofectamine 2000(Invitrogen, Carlsbad, CA, USA) according to the manufacturer’s instructions.

### Northern Blot Analysis

Total RNA was extracted from tumor cell lines using TRIzol regent (Invitrogen, USA) according to manufacturer’s instructions. Twenty micrograms of total RNA samples were denatured, size fractionated by electrophoresis in 1.2% agarose-formaldehyde gels, and transferred onto magna nylon transfer membrane (Osmonics Inc in Minnetonka, MN, USA). For the detection of endogenous CSTP1 mRNA, ORF of CSTP1 was excised from pcDNA3.1 myc/his C-CSTP1 plasmid, labeled with [α-32P]dCTP using Prime-a-Gene Labeling System (Promega, Madison, WI,USA). Internal control of GAPDH was obtained by PCR method with primers (GAPDH_NB_F and GAPDH_NB_R, Table 1) and was labeled as above. Hybridization was performed in ExpressHyb hybridization solution (Clontech, Mountain View CA, USA) according to the manufacturer’s instruction.

### In vitro Phosphatase Assay

The 293T cells were transfected with pcDNA3.1 myc/his C-CSTP1 plasmid by using calcium phosphate transfection method. After 48 h, cells were lysed in phosphatase storage buffer. The fusion protein CSTP1-His was purified by EZview Red HIS-Select HC Nickel Affinity Gel (Sigma, Ronkonkoma, NY, USA). Proteins were used for Phosphatase Assay using Serine/Threonine Phosphatase Assay System (Promega, Madison, WI, USA) according to manufacture’s instructions.

To examine whether purified CSTP1 could dephosphorylate Akt, CSTP1 was expressed as a GST fusion protein in BL21 (DE3) bacterial and purified with glutathione-Sepharose. The dephosphorylation reactions were carried out as described by Tianyan Gao [13].

### Baculovirus Vector Construction, Virus Packaging

To obtain His tagged Akt1, the full length coding region for Akt1 was first cloned into pcDNA3.1Myc/His C plasmid by PCR with primers Akt_His_F and Akt_His_R(Table 1), then, Akt1-His coding sequence was prepared by PCR method with primers Akt_Bac_F and Akt_Bac_R(Table 1) with pcDNA3.1Myc/His C-Akt1 plasmid as template. The product was subcloned into the BamHI and XhoI sites of pFastBacTM baculovirus shuttle vector (Invitrogen, Carlsbad, CA, USA). The recombinant pFastBacTM donor plasmid is transformed into DH10BacTM for transposition into the bacmid. White colonies contain the recombinant bacmid were selected for isolation of recombinant bacmid DNA. To generate viral particle, Sf9 cells in 6-well plates were transfected with the recombinant CSTP1 bacmid DNA in CellfectinTM(Invitrogen, Carlsbad, CA, USA). Five days later, medium was collected and the supernatant was reserved as P1 viral stock.

### Flow Cytometry Analysis

For apoptosis assay, cells were treated with 10^−8^ mol/L gemcitabine or 2 µg/mL cisplatin respectively. 48 hours later, cells were obtained and double stained with Annexin V-FITC and PI (KEYGEN, Nanjing, China). Cell apoptosis was analyzed by flow cytometry (BD Biosciences, San Jose, CA, USA).

For cell cycle analysis, cells were cultured in standard RPMI1640 with 10% FBS to approximately 40% confluencey, and cultured with 2 mM thymidine for 19 h. Cells were depleted of thymidine and incubated for 9 h until 2 mM thymidine was added for the second time, and the cells were cultured for an additional 16 h. After removal of thymidine again, the thymidine-synchronized cells were cultured in complete medium and collected at different times for cell cycle analysis. In general, cells were washed twice with PBS solution and fixed with chilled 70% alcohol at −20°C for 24 h. Cell sediment was collected by centrifugation, washed twice with PBS solution, incubated with 20 µl RNase A (20 mg/ml) for 30 min at 37°C, and stained with 25 µg/ml PI for 30 min at room temperature. Cell cycle distribution was then evaluated using flow cytometry(BD Biosciences,USA).

### Immunohistochemistry Analysis

Four-micrometer sections from formalin-fixed paraffin-embedded tissues were mounted on poly-L-lysine-coated slides and then deparaffinized in xylene and rehydrated through alcohol to distilled water. Endogenous peroxidase activity was blocked with 3% hydrogen peroxide for 15 minutes at room temperature. After pressure cooking the slides in 10 mM EDTA (pH 8.0) for 3 minutes, sections were incubated overnight at 4°C with rabbit anti-CSTP1 antibody (1∶200). Primary antibodies were detected using a two-step EnVision System (Dako, Denmark). Positive and negative immunohistochemistry controls were routinely used. For negative controls, the primary antibody was replaced by non-immune rabbit serum to confirm its specificity.

Evaluation of CSTP1 staining was principally according to the scoring criteria described previously [17]. The immunohistochemical quantitative reference scale ranging from 0 to 3 depended on the intensity of CSTP1 protein expression. The relative amount of tumor cells that stained positively for CSTP1(0–100%) in conjunction with the rating of the staining intensity, resulted in a staining score ranging from 0 to 100.

### Statistical Analysis

Recurrence-free survival was evaluated by Kaplan-Meier curves. Differences between the groups were evaluated by the log rank test. Disease-free survival was defined as the period between surgery and the detection of initial local recurrence. All analysis was performed by statistical software SPSS 12.0 and P value less than 0.05 was considered statistically significant.

## Results

### CSTP1 mRNA Expression in Normal and Tumour Tissues and Cell Lines

**Figure 1:**
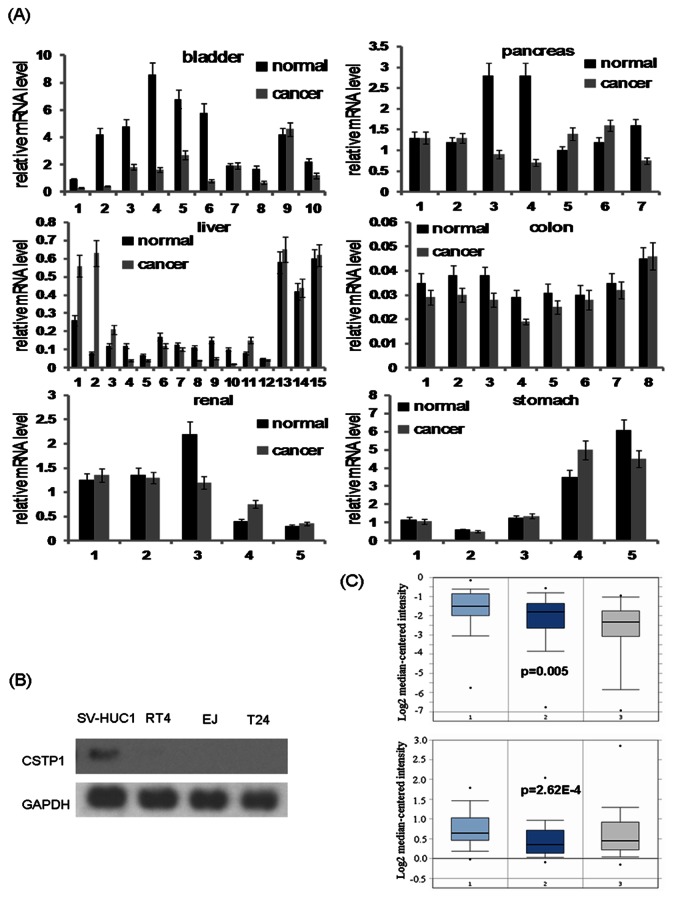
CSTP1 mRNA is down-regulated in bladder cancer tissues and cell lines. (A) Real-time PCR analysis of *CSTP1* mRNA levels in six kinds of human cancer tissues. *CSTP1* mRNA was selectively down-regulated in bladder cancer tissues compared to adjacent non-cancerous tissues. (B) Northern blot analysis of CSTP1 mRNA in bladder cancer cell lines. *CSTP1* mRNA was expressed at a moderate level in SV-HUC1 cells, but could be hardly detected in RT4, EJ and T24 bladder cancer cell lines, GAPDH was used as internal control. (C) CSTP1 mRNA expression analysis on Oncomine. Decreased CSTP1 mRNA expression in bladder cancers in two sets of dataset, with p-values of 0.005(up) and 2.62E-4(down) respectively.(1, bladder mucosa; 2, infiltrating bladder urothelial carcinoma; 3, superficial bladder cancer).

*CSTP1* gene was first identified by Bai GQ in 2005 [15], which was transactived by complete S protein of hepatitis B virus, and we supposed it may be associated with human cancers. We first examined *CSTP1* mRNA expression level in several kinds of human cancers, including liver, pancreas, stomach, colon, bladder and renal cancers. To our surprise, *CSTP1* mRNA was decreased significantly (by 40%∼90%) in 80% (8 of 10) of bladder cancer tissues as compared to paired adjacent non-cancerous tissues, whereas the expression of *CSTP1* mRNA in liver, pancreas, stomach, colon and renal cancer tissues did not change significantly (Fig. 1A). We then determined the expression level of CSTP1 mRNA by Northern blot in bladder cancer cell lines and SV-HUC1, a non-transformed urothelial cells. Results of Northern blot shown a moderate expression level of *CSTP1* mRNA in SV-HUC1 cells, but in RT4, EJ and T24 bladder cancer cells, *CSTP1* mRNA could hardly be detected(Fig. 1B). Furthermore, two sets of microarray dataset obtained from Oncomine also revealed that CSTP1 mRNA expression decreased in bladder cancers, with p-values of 0.005(up) and 2.62E-4(down) respectively(Fig. 1C).

### Characterization of CSTP1

**Figure 2:**
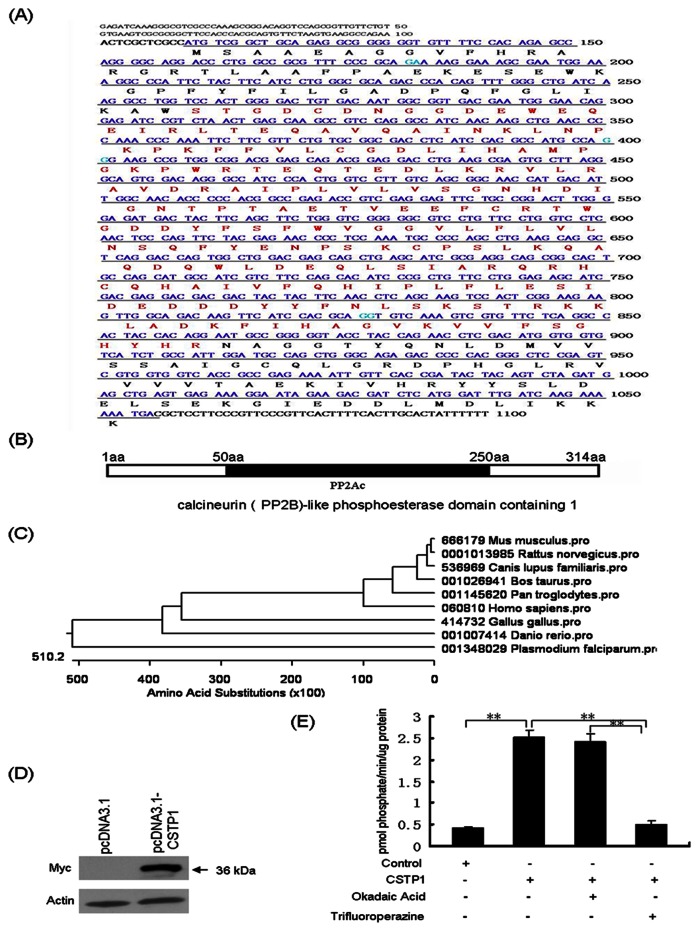
Characterization of CSTP1. (A) the nucleotide sequence of *CSTP1* open reading frame and the deduced amino acid sequence. The nucleotide sequence is shown in the 5' to 3' direction. The predicted amino acid sequence is shown below the nucleotide sequence. The amino acid residues corresponding to the phosphorase 2A catalytic unit domain (PP2Ac) are in red letters. (B) Bioinformatics analysis indicated that CSTP1 protein contained a conserved PP2Ac domain between 50 to 250 amino acids. (C) Phylogenetic analysis showed that CSTP1 protein is conserved in chimpanzee, dog, cow, mouse, rat, chicken, zebrafish, and P.falciparum. (D) Western blot analysis of CSTP1 expression in HeLa cells. HeLa cells were transfected with pCDNA3.1 Myc/His C-CSTP1 plasmid, 48 h later, the level of CSTP1-Myc fusion protein was tested by anti-Myc antibody. Actin was used as internal control. (E) CSTP1 displayed a PP2B-like protein phosphatase activity *in vitro*. 293T cells were transfected with pcDNA3.1Myc/His C-CSTP1, 48 h later, the CSTP1-His fusion protein was purified for phosphatase assay. The release of Pi was determined in the presence of 1 mM EGTA, 50 mM MgCl_2_, 5 mM NiCl_2_ and 250 µg/ml calmodulin. 200 nM trifluoroperazine or 0.5 µmol of Okadaic Acid was also added to abrogate the phosphatase activity of PP2B or PP2A.

Bioinformatics analysis showed that CSTP1 mRNA is 6156 bp in length, encoding a protein of 314 amino acids (Fig. 2A). The predicted molecular mass of this protein is 36 kDa with the theoretical isoelectric point of 5.99. The corresponding gene was mapped to chromosome 16p13.12, consisting of four exons and three introns. Sequence analysis of the predicted protein showed that the CSTP1 protein contains a PP2Ac(protein phosphatase 2A catalytic unit) domain from amino acid 50 to 250 (Fig. 2B). Phylogenetic analysis indicated that the CSTP1 gene is conserved among chimpanzee, dog, cow, mouse, rat, chicken, zebrafish, and P.falciparum (Fig. 2C).

Next, we confirmed the predicted molecular weight of CSTP1 protein. The pcDNA3.1Myc/His C-CSTP1 plasmids were transfected into HeLa cells and CSTP1-Myc fusion protein was determined by western blot with anti-Myc antibody. As shown in Fig. 2D, the recombinant plasmid expressed a protein with the expected molecular weight of 36 kDa.

Finally, we determined if CSTP1 protein displayed a Serine/Threonine phosphatase activity in vitro. 293T cells were transfected with pcDNA3.1myc/his C-CSTP1 plasmids and the CSTP1-His fusion protein were purified for phosphatase assay. The enzymatic activity was determined by measuring the release of Pi from the commercial synthetic peptide substrate containing a phosphothreonine residue [RRA(pT)VA]. As shown in Fig. 2E, CSTP1 protein catalyzed Pi released from RRA(pT)VA peptides, furthermore, PP2B specific inhibitor, trifluoroperazine almost abrogated its phosphatase activity, while PP2A specific inhibitor Okadaic Acid did not affect CSTP1 activity.

### Subcellular Localization of CSTP1

**Figure 3:**
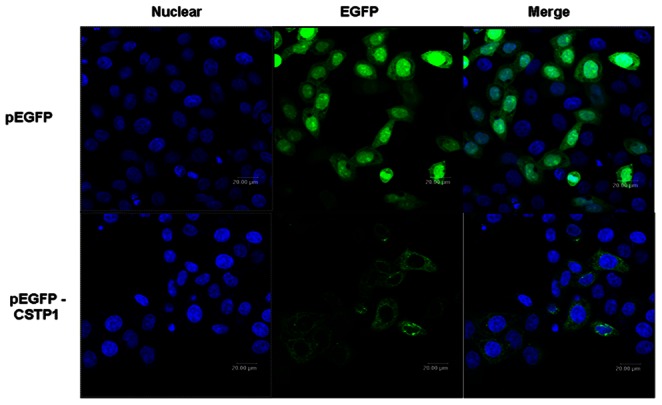
Cellular localization of CSTP1 protein. Hela cells were transfected with pEGFPN1 and pEGFPN1-CSTP1 plasmid respectively, 48 hours later, cells were fixed with 4% paraformaldehyde and visualized by fluorescence confocal microscopy. 4, 6-Diamidino-2-phenylindole dihydrochloride staining was used to indicate the cell nucleus. Results were representative of three independent experiments.

To gain insight into the biological function of CSTP1 protein, we first analyzed its subcellular localization. GFP-tagged CSTP1 expression plasmids were transfected into HeLa cells, and the fluorescence was visualized under fluorescence microscope. Cells transfected with pEGFP empty vector displayed diffuse fluorescence throughout subcellular compartments of the cells, while CSTP1-GFP mainly localized to the cytoplasm (Fig. 3), suggesting that CSTP1 may exert it’s function through localization to cell cytoplasm.

### CSTP1 Over-expression Suppresses Bladder Cancer Cell Proliferation and Colony Formation, but not Invasion

**Figure 4:**
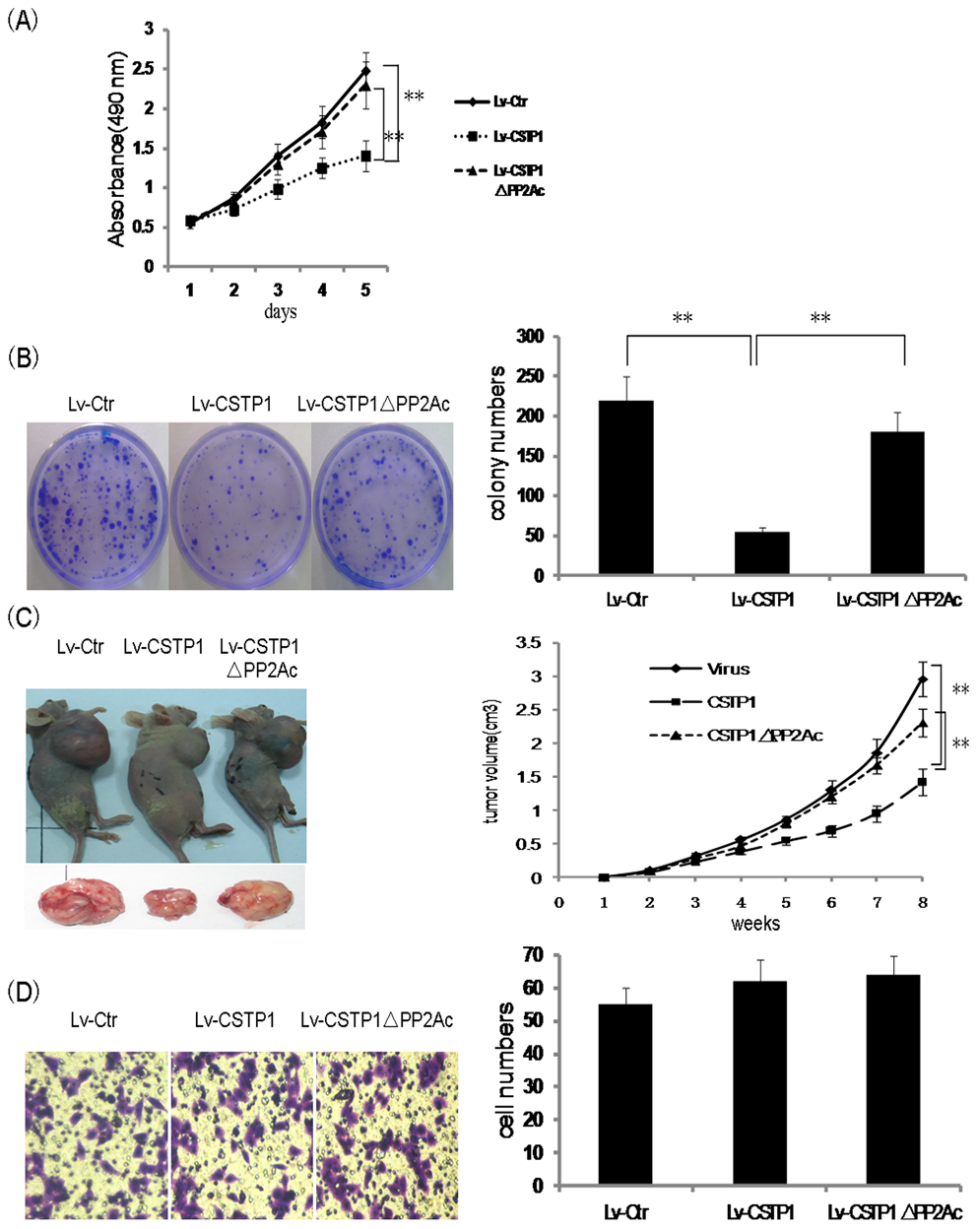
Over-expression of CSTP1 suppresses bladder cancer cells proliferation. (A) EJ cells were transduced with lentiviruses overexpressing CSTP1 (Lv-CSTP1), the PP2Ac domian deleted CSTP1 (Lv-CSTP1 ΔPP2Ac), or lenti-vector control (Lv-ctr). Cell proliferation was detected by MTT assay. Data at each time point represents the mean ± SD of 8 samples. Results were representative of three independent experiments. (B) The transduced EJ cells were cultured for 14 days, and colonies were stained using crystal violet and counted within the field of a ×40 objective lens. Results were representative of three independent experiments.(C) The xenograft tumor growth in nude mice were remarkably suppressed following CSTP1 overexpression. Data at each time point represents the mean ± SD, n = 6. (D) Chamber assays were performed with transduced EJ cells. The results showed that CSTP1 has no effect on cell invasion . Data shown were mean number of invading cells (×100 field) ± SD from three independent experiments. **, p<0.01.

Given the observation that CSTP1 mRNA was decreased in bladder cancer tissues, we presumed that CSTP1 may function as a tumor suppressor in bladder cancers. To investigate the role of CSTP1 in cell function, we first analyzed the effect of CSTP1 overexpression on cell proliferation. CSTP1 was stably overexpressed in EJ bladder cancer cells via lentiviral infection and cell proliferation was assessed by MTT method. Overexpression of CSTP1 inhibited the proliferation of EJ cells by 50% after a 5-days’ culture (Fig. 4A) , while, depletion of PP2Ac domain impaired the growth-inhibition ability of CSTP1 on EJ Cells by 80%( Fig. 4A). Consistent with the results of MTT assay, overexpression of CSTP1 decreased the colony formation capacity of EJ cells, and depletion of PP2Ac domain rescued the colony formation ability of EJ cells (Fig. 4B). Similar results were obtained in another bladder cancer cell line T24 (data not shown).

To investigate the role of CSTP1 in vivo, we established human bladder xenograft tumors in nude mice by injection of control EJ cells or EJ cells stably expressing either wild type CSTP1 or CSTP1 ΔPP2Ac. Growth of the implanted tumors was measured in mice (n = 6 for each group) over a period of 8 weeks. Results indicated that, in athymic mice receiving the EJ cells overexpressing CSTP1, tumor growth was significantly suppressed(∼50%), but in athymic mice receiving EJ cells overexpressing CSTP1 ΔPP2Ac, tumor growth almost did not change compared to the control group(Fig. 4C). However, CSTP1 overexpression had no effect on EJ cell invasion as determined by transwell assays (Fig. 4D).

### CSTP1 Influence on Cell Cycle and Apoptosis

**Figure 5:**
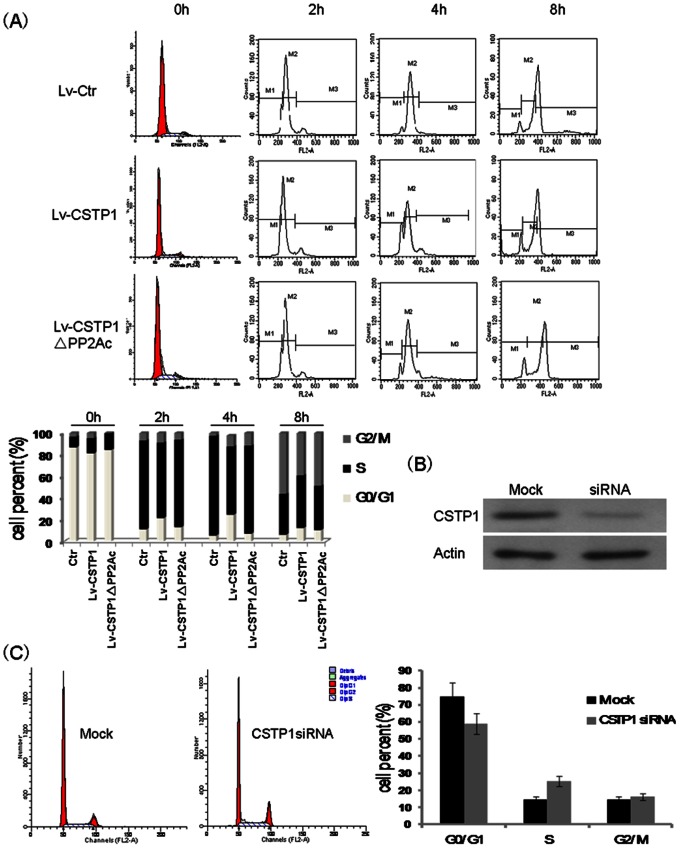
CSTP1 inhibits cell cycle progression. (A) EJ cells overexpressing CSTP1 (Lv-CSTP1), CSTP1 ΔPP2Ac (Lv-CSTP1 ΔPP2Ac) and control cells were treated with two rounds of 2 mM thymidine. Cell cycles were analyzed by FACS after releasing from G0/G1 phase at indicated time point. Results were representative of three independent experiments. (B) Immunoblotting of CSTP1 in extracts of SV-HUC1cells 48 h after transfection with a control siRNA (Mock) or a siRNA for CSTP1 (CSTP1 siRNA). (C) Cell cycle analysis of SV-HUC1 cells after transfection with control siRNA or siRNA for CSTP1. *,p<0.05. Results were representative of three independent experiments.

To explore the effect of CSTP1 on cell cycle, lentivirus transduced EJ cells were synchronized at G0/G1 phase by two rounds of thymidine treatment and cell cycle were analyzed by FACS at 0 h, 2 h, 4 h, and 8 h after releasing from G0/G1 phase by the addition of complete medium. As shown in Fig. 5A, overexpression of CSTP1 significantlly delayed the progression of cell cycle. Compared with the control cells, cells overexpressing CSTP1 exhibited a lower percentage of cells in S phase at 2 h and 4 h after releasing from G0/G1 phase. Furthermore, a lower percentage of G2/M phase cells at 8 h after released from cell cycle blocking were also observed in CSTP1-overexpressing cells, while cells overexpressing CSTP1 ΔPP2Ac did not show delayed cell cycle progression. To confirm the effects of endogenous CSTP1 on cell cycle, CSTP1 protein was knocked down by siRNA transfection in SV-HUC1 cells which showed a moderate CSTP1 expression (Fig. 1B). CSTP1 protein was effectively down-regulated by specific siRNA transfection (Fig. 5B), and the reduced expression of CSTP1 promoted cell proliferation by elevating the percentage of cells in S phase (Fig. 5C).

**Figure 6:**
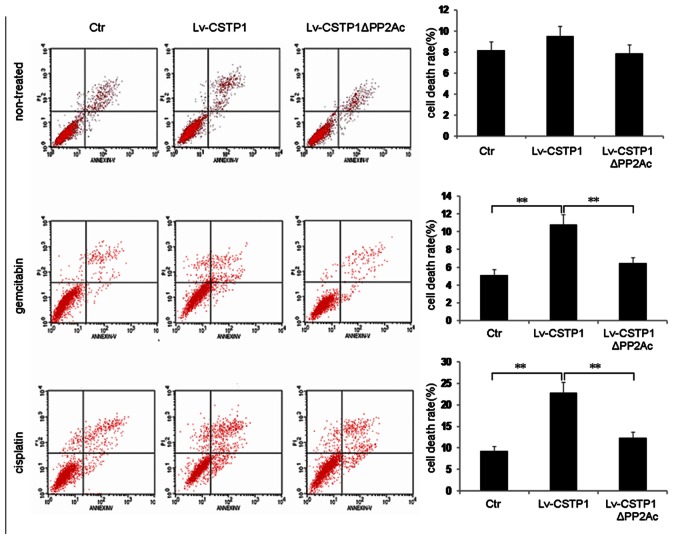
CSTP1 promotes cell apoptosis. EJ cells stably over-expressing CSTP1 or CSTP1 ΔPP2Ac and control cells were cultured in complete medium with or without gemcitabine(10^−8 ^mol/L) and cisplatin(2 µg/mL), 48 hours later, cells were double stained with annexin V-FITC and PI, cell apoptosis was analyzed by FACS. Results were representative of three independent experiments. **, p<0.01.

To examine the role of CSTP1 on cell apoptosis, EJ cells overexpressing CSTP1 or CSTP1Δ PP2Ac and control cells were cultured in complete medium with or without gemcitabine and cisplatin, two commonly used chemotherapy drugs in bladder cancer treatment. The FACS results shown that overexpression of CSTP1 alone only slightly induced EJ cells apoptosis, but when the cells were treated with chemotherapy drugs, an increased death rate was observed in CSTP1-overexpressing cells, and depletion of PP2Ac domain attenuated this death-promoting ability of CSTP1 dramatically (Fig. 6).

### CSTP1 Interacts with and Dephosphorylates pAkt at Ser473 Site

**Figure 7:**
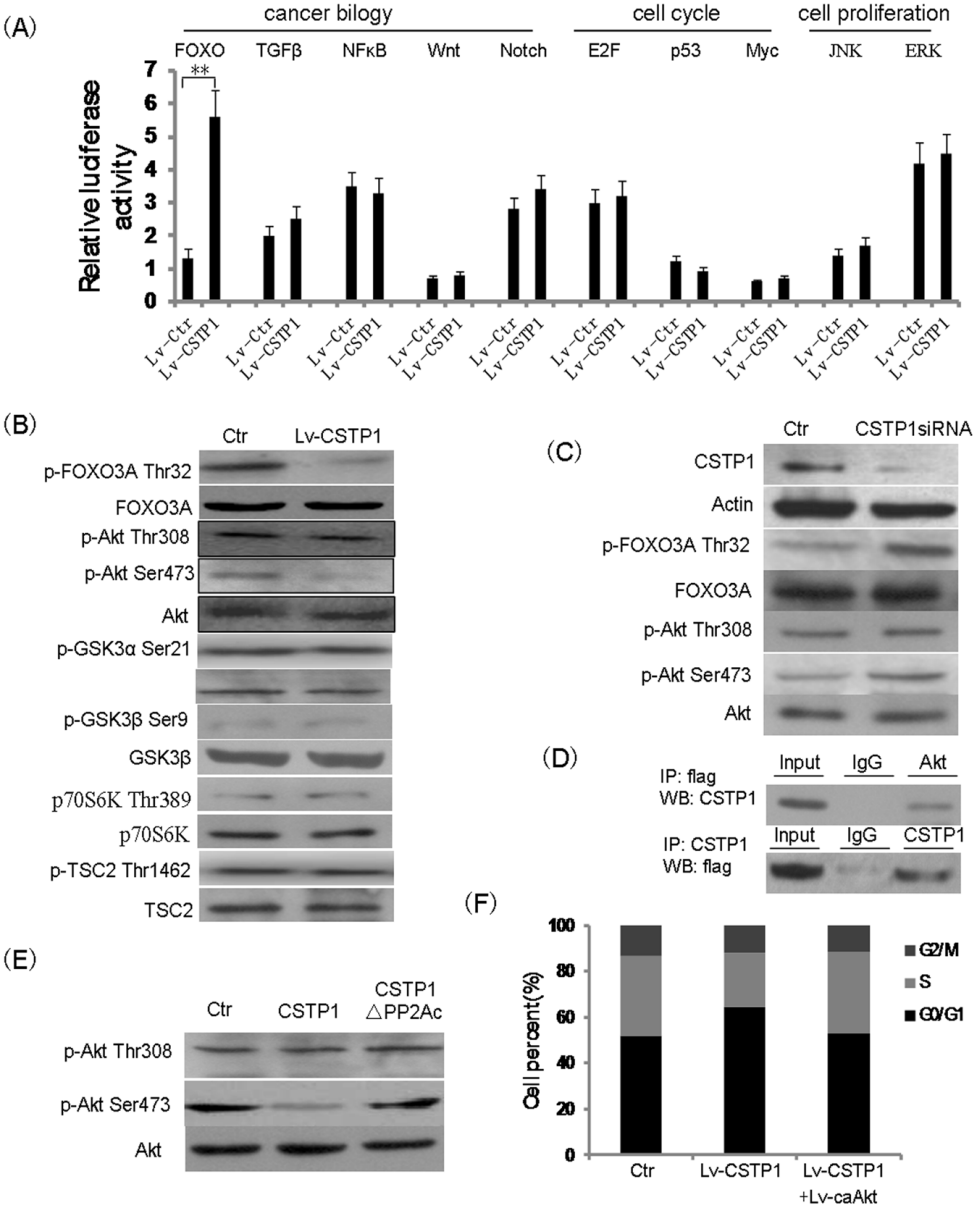
CSTP1 interacts with and dephosphorylates Akt at Ser473 site. (A) EJ cells overexpressing CSTP1 and control cells were transfected with a series of cignal reported plasmid, 48 h later, luciferase activity was measured using the luciferase assay system with a luminometer. luciferase-based reporter signal is normalized to the expression of a cotransfected Renilla luciferase control plasmid. (B) Western blot analysis of the phosphorylation levels of FOXO3A, Akt,GSK3α, GSK3β, p70S6K and TSC2 in EJ cells overexpressing CSTP1. Unphosphorylated FOXO3A, Akt, GSK3α, GSK3β, p70S6K and TSC2 proteins were detected as internal controls. (C) Western blot analysis of the phosphorylation levels of FOXO3A and Akt after knocking down of CSTP1 in SV-HUC1 cells. (D) CSTP1 interacts with Akt in 293T cells. 293T cells were cotransfected with pcDNA3.1-CSTP1 and Flag-Akt expressing plasmids, interaction between CSTP1 and Akt was analyzed by co-ip experiment with anti-Flag (up panel) or anti-CSTP1 antibody(low panel). (E) Dephosphorylation of Akt by purified CSTP1 *in vitro*. Pure His-Akt was incubated with GST-tagged CSTP1 or GST-tagged CSTP1ΔPP2Ac in the phosphatase buffer. For a negative control, CSTP1 protein was omitted from the reaction mixture(Ctr). (F) EJ cells were overexpressed CSTP1 (Lv-CSTP1) or CSTP1(Lv-CSTP1) plus phosphor-mimetic S473D construct of Akt (ca-Akt) by lentivirus, cell cycle was analyzed by FACS. All experiments were performed at least three times.

To explore the underlined mechanisms responsible for the elevated apoptosis and retarded cell cycle of bladder cancer cells mediated by CSTP1, we seek to find signaling pathways downstream of CSTP1 overexpression. Signaling pathways that usually involved in cancer biology, cell cycle control or cell proliferation were examined in CSTP1-overexpressing EJ cells by analysis of luciferase activity using Cignal Reporter Assay Kit. Results shown that FOXO factor had a more robust transcriptional activity in CSTP1-overexpressing cells(Fig. 7A). The increase of FOXO activity was further demonstrated by reduction in the phosphorylation state of FOXO3A at Thr32 site after CSTP1 overexpression (Fig. 7B). Since Akt kinase is the primary negative regulator upstream of FOXO factor, we supposed that Akt kinase activity could decreased in CSTP1-overexpressing cells. To confirm this hypothesis, the phosphorylated Akt at Thr308 or Ser473 site, which represents the activation state of Akt, were detected. Western blot results shown that overexpression of CSTP1 decreased the level of phosphorylated Akt at Ser473 site, but the level of phosphorylated Akt at Thr308 was unchanged (Fig. 7B). Since CSTP1 is downregulated in bladder cancer tissues, so we asked whether loss of CSTP1 expression in urothelial cells could result in the activation of Akt kinase and inactivation of FOXO factor. Consistent with the CSTP1 overexpression assay, knockdown of CSTP1 in SV-HUC1 increased the phosphorylation level of Akt at Ser473 site and FOXO3A at Thr32 site (Fig. 7C). To further investigate the effect of CSTP1 overexpression on the activity of Akt, we also examined other four well-known proteins that dependent on Akt for phosphorylation, GSK3α, GSK3β, p70S6K, and TSC2. To our surprise, CSTP1 overexpression did not affect the phosphorylation status of GSK3α, GSK3β, p70S6K, and TSC2 (Fig. 7B).

Next, we tested if CSTP1 could directly bind to and dephosphorylate phosphorylated Akt at Ser473. CSTP1 and Akt expression plasmids, harboring Myc and Flag tag respectively, were cotransfected into 293T cells. Total protein was extracted and immunoprecipitated with antibody against Flag tag, and western blots were performed using anti-CSTP1 antibodies. As shown in Fig. 7D, CSTP1 was co-immunoprecipitated with Flag-tagged Akt(up panel), the same result was obtained when the whole protein lysate was immunoprecipitated with anti-CSTP1 antibodies and immunobloted with anti-Flag antibodies(Fig. 7D, low panel). These results indicated that Akt interacts with CSTP1 in intact cells. To further explore whether purified CSTP1 can dephosphorylate Akt in vitro, full-length CSTP1 was expressed in bacteria as a GST fusion protein, and Akt dephosphorylation assay was performed as described by Tianyan Gao [13]. Purified CSTP1 fusion protein was incubated with pure baculovirus-expressed Akt which was phosphorylated on Thr308 and Ser473 [10], and western blot was performed to detect the protein level of Akt. As expected, CSTP1 decreased Ser473 phosphorylation level of Akt, but the phosphorylation state of Thr308 site was unchanged, suggesting that CSTP1 can directly dephosphorylate Akt at Ser473 site (Fig. 7E).

Finally, we tested whether dephosphorylation of Akt kinase at Ser473 site is required for the ability of CSTP1 to suppress cell cycle progression. EJ Cells overexpressing CSTP1 were co-infected with lentivirus-Akt(S473D), a constitutively active phosphor-mimetic Akt(ca-Akt)[18], [19], cell cycle was analyzed by FACS. As shown in Fig. 7F, overexpression of ca-Akt effectively rescued the CSTP1-dependent cell cycle arrest.

### Immunohistochemical Analysis of CSTP1 in Paraffin Embedded Tissue and Survival Analysis

**Figure 8:**
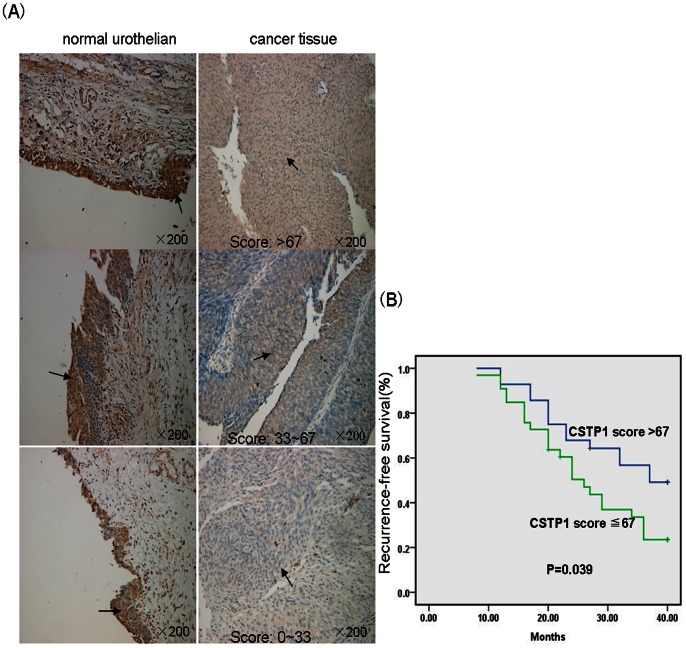
Decreased CSTP1 protein expression is correlated with tumor recurrence in patients with non-muscular invasive bladder cancer. (A) Representative immunohistochemistry staining of CSTP1 protein in formalin-fixed paraffin-embedded tumor or normal urothelia tissues. (B) Recurrence-free survival was analyzed by Kaplan-Meier method. The expression level of CSTP1 in bladder cancer tissues positively correlated with the recurrence-free survival in patients suffered from non-invasive bladder cancers. p = 0.039, Log-rank test.

To explore the clinical relevance of CSTP1 with bladder cancer, immunohistochemical staining of CSTP1 was performed in tissues from a cohort of 86 bladder cancer patients. Of the 86 patients, 62 were in stage pTa and pT1 (non-invasive), and the rest 24 were in pT1∼ pT4 (muscle-invasive). Immunostaining scores were estimated as described in methods. Decreased CSTP1 staning was detected in all the pTa and pT1 tumor tissues compared to adjacent non-cancerous tissues, while only 10 of the 26 cases of muscle invasive bladder cancer tissues shown a decreased staining of CSTP1, no change was observed in 11 of the muscle invasive bladder cancer tissues, and the remaining 5 showed an increased staining of CSTP1, the representatives of CSTP1 staining in pTa and pT1 bladder cancer tissues were presented in Fig. 8A.

Because of the limited data acquired from muscle invasive patients, the effects of the decreased expression of CSTP1 on the clinical outcomes were examined only in non-invasive bladder cancer patients. Follow-up data for at least 40 months following transurethral resection of the primary lesion were collected for all non-muscle invasive tumor patients. Patients were classified into two groups according to the characteristics of the immunohistochemical staining (group A, staining score >67; group B, staining score = 0–66). As shown in Fig. 8B. the duration of the recurrence-free survival time was significantly decreased in patients from group B (the median survival time for patients from group A and B were 37 and 26 months, respectively) (p = 0.039; Log-rank test).

## Discussion

Just as in many other types of cancers, Akt kinase is often aberrantly activated in bladder cancers [2]. Activated Akt kinase plays a pivotal role in controlling cell growth, proliferation and survival [20]. Several molecular mechanisms have been identified to aberrantly activate the Akt kinase signaling pathway in bladder cancers, including: (1) mutations of upstream activators, such as p110α (PIK3CA), FGFR3 and Ras[21]–[24]; (2) activating mutations of the Akt kinase [25], and (3) loss of heterozygosity (LOH), homozygous deletion and inactivating mutations of the negative regulator PTEN[26]–[29]. Although great progress has been made in elucidating the mechanisms leading to Akt phosphorylation and activation, little is known about the mechanisms terminating Akt signaling. In 2005, a model was proposed by Tianyan Gao [13] that the termination of Akt signaling was controlled by two key proteins, PTEN and PHLPP. PTEN is a lipid phosphatase that prevents activation by removing the second messenger that activates Akt. PHLPP is a protein phosphatase that inactivates Akt by direct dephosphorylation of Ser473 site in the hydrophobic motif. In this study, we identified a novel protein phosphatase, CSTP1, which catalyzed dephosphorylation of phosphothreonine residue [RRA(pT)VA] in vitro, and PP2B specific inhibitor can abrogated its phosphatase activity, suggested that CSTP1 protein displayed a PP2B-like phosphatase activity. In vitro and in vivo experiments demonstrated that CSTP1 can interact and selectively dephosphorylate Akt at S473 site, suggesting a possibility that each phosphorylation sites on Akt can be targeted by more than one phosphatase.

It has been demonstrated that many oncogenes and proto-oncogenes are phosphorylated protein kinases [30], [31]. In most cases, their activation contributes to tumorigenesis, therefore, protein phosphatases which exhibite the opposite activity of kinases may serve as tumor suppressors, with the PP2A protein phosphatase as an example [32]. Consistent with this knowledge, our study showed that the expression of CSTP1 mRNA dramatically decreased in 80% of bladder cancer tissues. Overexpression of CSTP1 inhibited bladder cancer cell proliferation, colony formation in vitro and bladder xenograft tumor growth in nude mice. This suggests that protein phosphatase CSTP1 could function as a tumor suppressor, at least, in human bladder cancers. Blocking cell cycle progression and inducing apoptosis are two important mechanisms for anti-proliferative gene to suppress cancer cells growth. Cell cycle and apoptosis analysis revealed that CSTP1 overexpression inhibited cancer cells to go through the cell cycle and sensitized bladder cancer cells to chemotherapy drugs, and depletion of PP2Ac domain abrogated the growth-inhibition and death-promotion ability of CSTP1, suggesting that CSTP1 exerted its tumor suppression function through inhibiting cell cycle progression and promoting cell apoptosis., and that protein phosphatase activity of CSTP1 plays a crucial role in its tumor suppression activity. Expression profile analysis revealed that CSTP1 mRNA was selectively decreased in non-invasive bladder cancer tissues, suggesting that CSTP1 plays an important role mainly in bladder carcinogenesis, and it may be used as a potential target for bladder cancer treatment.

Since complete activation of Akt requires the phosphorylation at both Thr308 and Ser473 [33] site, and phosphorylation at Ser473 appears to be critical for the regulation of Akt activity because selective dephosphorylation of Thr308 does not significantly affect the activity of Akt [34]. Moreover, our results showed that Ser473 dephosphorylation of Akt by CSTP1 did not affect the phosphorylation levels of Akt targets GSK3, p70S6K and TSC2, although the phosphorylation level of FOXO3A could be regulated. This was in accordance with published data reported by Jacinto [35] that defective Ser473 phosphorylation affected only a subset of Akt targets in vivo, including FOXO1/3A, while other Akt targets, TSC2 and GSK3, and the TORC1 effectors, S6K and 4E-BP1, were unaffected. Therefore, dephosphorylation at S473 determines the specificity of Akt’s function rather than its absolute activity.

Bladder cancers can be divided into two major groups: non-invasive papillary tumors and muscle-invasive tumors. In the majority of cases, these two groups of carcinomas do not represent a developmental continuum [36], [37] , and are thought to arise from at least 2 separate mechanisms. Muscle-invasive bladder cancer (T2–T4) is thought to develop via flat dysplasia and carcinoma in situ(CIS) and these tumours are usually of non-papillary architecture. Non-invasive(Ta) tumours are believed to arise via flat urothelial hyperplasia followed by development of an exophytic papillary architecture [2]. A more prevalent decrease of CSTP1 expression was found in non-invasive bladder cancer tissues than that in the muscle-invasive tissues, provides a new strong evidence to this point of view. The major problem in the clinical treatment of non-muscle invasive bladder is the frequent recurrence. Much research has focused on the identification of the molecular or epigenetic markers to precisely predict the clinical outcome of non-muscle invasive bladder cancer, but little progress has been made. In our study, expression levels of CSTP1 are positively correlated to the recurrence-free survival of patients with Ta and T1 bladder cancers, indicate it may be a useful marker for prognostic prediction of non-muscle invasive bladder cancers.

Taken together, we have identified a novel protein phosphatase targeting Akt protein kinase, CSTP1, which dephosphorylates the hydrophobic motif of Akt specifically at the Ser473 site, blocks cell cycle progression, promotes cell apoptosis and suppresses tumor growth in nude mice. CSTP1 expression was selectively reduced in bladder cancer tissues and its expression level is positively correlated to the recurrence-free survival of patients with Ta and T1(non-invasive) bladder cancers, and may serve as a prognostic marker for non-muscle invasive bladder cancers.
